# Flow molecular dynamics simulations reveal mechano-presentation of von Willebrand factor through glycan-modulated autoinhibitory modules

**DOI:** 10.1063/5.0329754

**Published:** 2026-05-13

**Authors:** Naveen Eugene Louis Richard Louis, Yunduo Charles Zhao, Lining Arnold Ju

**Affiliations:** 1 School of Biomedical Engineering, The University of Sydney, Darlington, NSW 2008, Australia; 2 Charles Perkins Centre, The University of Sydney, Camperdown, NSW 2006, Australia; 3 The University of Sydney Nano Institute (Sydney Nano), The University of Sydney, Camperdown, NSW 2006, Australia; 4 Heart Research Institute, Newtown, NSW 2042, Australia

## Abstract

Force-induced protein conformational changes govern many essential biological processes, yet their molecular mechanisms remain difficult to resolve. Von Willebrand factor (VWF), a central regulator of hemostasis, is activated by hydrodynamic forces in blood flow, but how mechanical signals propagate across its multidomain architecture is poorly understood. Here, we use flow molecular dynamics (FMD), a simulation framework that applies fluid forces via controlled solvent flow to interrogate mechanosensitive proteins. Using VWF as a model system, we reconstructed the complete mechanomodule (D′D3–A1–A2–A3; 1110 residues) with native glycosylation by integrating crystallographic data and ColabFold predictions. FMD simulations capture a force-driven transition from a compact, autoinhibited “bird's nest” ensemble to an extended, activated state, revealing asymmetric autoinhibitory strengths within the N′AIM and C′AIM modules of the A1 domain. By directly linking static structures to dynamic, force-regulated behavior, this work establishes a generalizable platform for dissecting protein mechanosensitivity and enabling the rational design of force-responsive therapeutics.

## INTRODUCTION

**FIG. 1.**
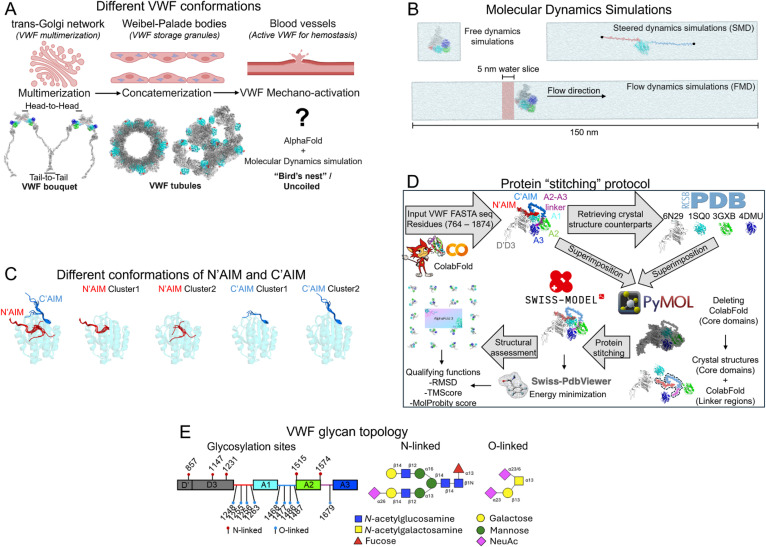
Conformational transitions underlying mechano-presentation of VWF and overview of the molecular modeling workflow and structural organization of the VWF mechanomodule. (a) VWF monomers assemble into ultra-large multimers within the trans-Golgi network through head-to-head dimerization mediated by the D′D3 domains and tail-to-tail interactions at their C-terminal cystine knot regions. These multimers are densely packed as helical tubules in Weibel–Palade bodies (WPBs) for intracellular storage. Upon vascular injury, WPBs undergo exocytosis, releasing VWF into the circulation, where the helical tubules unfurl under shear flow to expose platelet-binding sites, initiating platelet adhesion and thrombus formation. (b) Representative simulation strategies employed in this study, including equilibrium (free), flow-conditioned, and steered molecular dynamics, used to interrogate VWF mechanoactivation pathways. (c) Structural clustering of all available crystal structures of the A1 domain, illustrating conformational diversity in the N-terminal AIM (N′AIM) and C-terminal AIM (C′AIM) motifs (N′AIM Cluster1: 1IJB, 7EOW, 1UON, 5BV8, 1AUQ, 1UEX, 1IJK; N′AIM Cluster2: 1FNS, 1SQ0, 1OAK, 7F49, 1M10, 4C2B, 3HXO, 4C29, 3HXQ, 4C2A; C′AIM Cluster1: 1SQ0, 4C2A, 1M10; C′AIM Cluster2: 1IJB, 7EOW, 1UON, 5BV8, 1AUQ, 1UEX, 1IJK, 1FNS, 1OAK, 7F49, 4C2B, 3HXO, 4C29, 3HXQ). (d) Protein reconstruction strategy combining ColabFold-based predictions for unresolved linker regions with homology modeling guided by existing crystal structures. This integrative approach enables high-resolution atomic modeling of the VWF mechanomodule. (e) Left: Mapping of *N*- and *O*-linked glycosylation sites across the VWF sequence, highlighting their spatial distribution and occupancy. Right: Representative glycan topology used in simulations.

Von Willebrand factor (VWF) exemplifies a finely tuned mechanosensitive protein system in which force-dependent conformational transitions directly govern its essential role in hemostasis.^1,2^ Under physiological flow, VWF adopts a compact, globular “bird's nest” conformation that conceals its platelet-binding domains, thereby preventing spontaneous adhesion.^3,4^ However, at sites of vascular injury, elevated shear stress acts as a mechanical cue that triggers VWF to unfurl into an extended conformation—mechano-presentation^5^—exposing cryptic binding sites for the platelet surface receptor glycoprotein Ibα (GPIbα).^6^ Remarkably, the spatial organization of VWF is highly context dependent. Within the trans-Golgi network, VWF monomers assemble via head-to-head interactions through the D′D3 domains and tail-to-tail associations via their C-terminal regions, forming higher-order multimers with a characteristic bouquet-like architecture^7^ [Fig. 1(a), left]. These multimers are compacted and stored within helical tubules inside Weibel–Palade bodies (WPBs), specialized endothelial granules that maintain VWF in a densely packed, secretion-ready state^8^ [Fig. 1(a), middle]. Upon vascular injury, WPBs undergo rapid exocytosis, releasing VWF into the bloodstream, where these tubules unfurl into elongated multimers that tether platelets and initiate thrombus formation^9^ [Fig. 1(a), right]. Although the functional consequences of this structural transition are well-established, the precise molecular architecture and mechanics governing VWF's force-induced unfurling remain poorly understood, representing a frontier in vascular mechanobiology.

Recent breakthroughs in AI-guided structural biology, exemplified by AlphaFold 3, have revolutionized our ability to visualize complex protein architectures.^10^ These tools provide unparalleled insights into static conformations and offer unprecedented opportunities to explore large, multidomain systems such as VWF. However, their static predictions limit their ability to capture force-induced transitions and dynamic rearrangements. To overcome these limitations, we subjected AI-derived models to a suite of molecular dynamics (MD) simulations [Fig. 1(b)], enabling us to explore the full mechanical landscape of VWF from its compact, autoinhibited conformation to the force-activated state required for platelet adhesion.

The compact, autoinhibited conformation of VWF represents a state of functional stasis,^7^ maintained through a hierarchical network of autoinhibitory mechanisms. The A1 domain central to platelet recruitment is subjected to two key levels of inhibition: (i) interdomain shielding by flanking domains such as the D′D3^11^ and A3 domain^12^ and (ii) intermodular autoinhibition by the N- and C-terminal autoinhibitory modules (N′AIM, residues 1238–1271, and C′AIM, residues 1459–1493), which sterically occlude the GPIbα-binding interface.^4^ However, the structural basis of this autoinhibition has remained elusive due to technical limitations in crystallography. Most AI-based prediction tools, such as AlphaFold, are trained on existing high-resolution crystal structures and thus struggle to accurately model flexible or unresolved regions such as the N′AIM and C′AIM modules, which are absent from all currently deposited A1 crystal structures [Fig. 1(c)]. We employed a hybrid strategy that combines deep learning-based protein structure prediction with homology modeling, yielding a more complete and functionally relevant representation of the autoinhibited VWF ensemble [Fig. 1(d)]. Furthermore, experimental efforts to produce AIM–A1 constructs with homogeneous *O*-glycosylation and resolve them at high resolution have been unsuccessful.^13^ Given emerging evidence that glycosylation modulates VWF mechanosensitivity and spatial conformation^14,15^ [Fig. 1(e)], our study incorporates *N*- and *O*-linked glycans associated with the VWF mechanomodule. This comprehensive modeling enabled us to explore how glycan-mediated shielding influences A1 accessibility. We also corroborate prior experimental evidence for the regulatory roles of N′AIM and C′AIM, while providing structural insight into why N′AIM exerts a more dominant autoinhibitory effect. Detailed steric-clash analyses mechanistically explain the differential binding affinities of distinct VWF constructs to GPIbα. Together, these findings refine our understanding of VWF mechanoregulation and establish a framework for mechanomedicine design,^16^ by delineating which VWF epitopes are shielded under static conditions and selectively exposed under force, i.e., mechano-presentation mapping.^5^

## RESULTS

### Hybrid modeling and simulation of VWF reveals high-confidence autoinhibited structure

The structural elucidation of glycoproteins such as VWF remains profoundly challenging, owing to the intrinsic chemical heterogeneity and dynamic conformational landscape imposed by their glycan moieties.^17^ These complexities have significantly hindered efforts to resolve extended segments of VWF through crystallography. This limitation is reflected in the PDB, where nearly all deposited VWF structures comprise either isolated domains or discontinuous fragments, frequently omitting critical interdomain linker regions. Notably, the longest crystallized N′AIM in PDB entry 1U0N^18^ only extends from Asp1260, thereby excluding the structurally unresolved but functionally essential distal segment spanning residues 1238–1259. Similarly, the most extended crystallized C′AIM captured to date PDB 5BV8^19^ terminates at Leu1469, omitting the distal residues 1470–1493 that are vital to understanding A1 domain regulation in its native context. Our integrative approach of modeling the hybridized model [ColabFold + SWISS-MODEL (CF + SM)] consisting of crystallographic cores and ColabFold-modeled linkers spanning from the D′D3–A3 domain achieved better scores for metrics such as root mean square deviation (RMSD) and TM-scores against crystal structures (supplementary material Table S1) and MolProbity scores following 500 ns simulations (supplementary material Table S2). Our rationale for focusing on the VWF mechanomodule arises from the observation that predictive models of the N′AIM–A1–C′AIM assembly, in the absence of flanking domains D′D3, A2, and A3, yield linker regions in an artificially extended, linear conformation [supplementary material Fig. S1(A)]. Moreover, each structural prediction results in a single, static representation of the mechanomodule [supplementary material Fig. S1(B)], failing to capture the dynamic conformational landscape intrinsic to VWF. These limitations underscore the necessity of integrating molecular dynamics (MD) simulations with structural predictions to resolve the full range of conformational transitions exhibited by VWF, both in the absence and presence of external mechanical forces [supplementary material Fig. S1(C)]. We further validated our model by closely examining the N′AIM–A1–C′AIM region to assess whether the orientation of the flanking autoinhibitory modules aligns with known structural data [supplementary material Fig. S2(A)]. Specifically, we compared our models against the A1 domain crystal structure (PDB ID: 1U0N), which contains the longest experimentally resolved N′AIM segment. The top-scoring AlphaFold 3 model and our CF + SM model both recapitulated similar poses for the structured portions of N′AIM (residues 1261–1271) and C′AIM (residues 1458–1468). However, notable variability persisted in the more flexible regions of N′AIM (residues 1238–1260) and C′AIM (residues 1469–1493). To determine which conformations may represent physiologically relevant autoinhibited states, we superimposed each model onto the A1–GPIbα complex structure (PDB ID: 1SQ0). This allowed us to evaluate the extent of steric hindrance imposed by N′AIM and C′AIM on the GPIbα-binding site. The top-scoring AlphaFold 3 model [supplementary material Fig. S2(B)] showed minimal obstruction of the GPIbα interface. In contrast, our CF + SM [supplementary material Fig. S2(C)] and glycosylated CF + SM [supplementary material Fig. S2(D)] models exhibited pronounced N′AIM-mediated shielding, consistent with the structural features expected of the compact, autoinhibited “bird's nest” conformation.

### Glycosylation modulates VWF mechanomodule assembly and dynamics

**FIG. 2.**
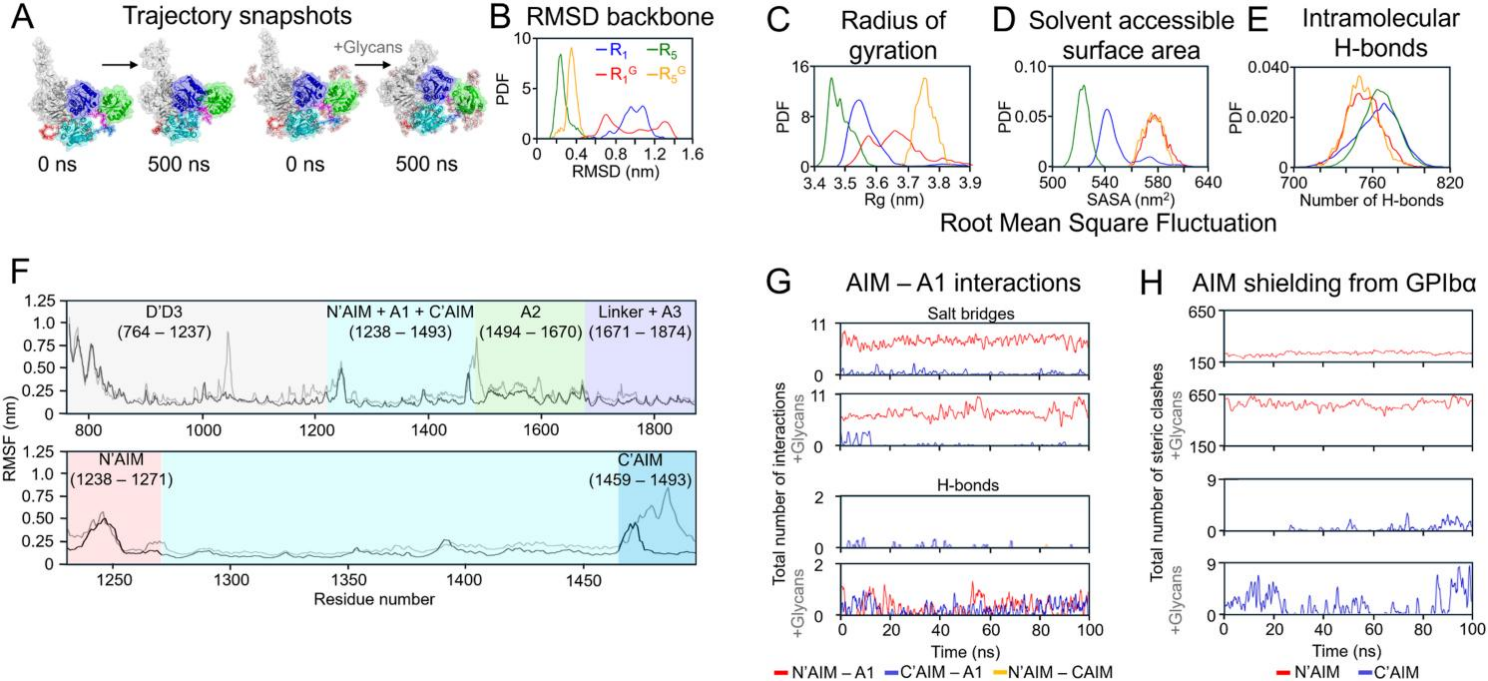
Free molecular dynamics simulations reveal the compact “bird's nest” conformation of VWF and the role of glycans in modulating structural dynamics. (a) Representative trajectory snapshots at t = 0 ns and t = 500 ns for glycosylated and non-glycosylated VWF mechanomodules, showing that glycans increase the overall hydrodynamic size. Probability density distributions comparing glycosylated and non-glycosylated mechanomodules across structural metrics: (b) backbone root mean square deviation (RMSD), (c) radius of gyration (Rg), (d) solvent-accessible surface area (SASA), and (e) number of intramolecular hydrogen bonds. Key: Blue: R_1_ (0–100 ns, no glycans); red: $R_{1}^{G}$ (0–100 ns, with glycans); green: R_5_ (400–500 ns, no glycans); orange: $R_{5}^{G}$ (400–500 ns, with glycans). (f) Root mean square fluctuations (RMSF) of Cα atoms across the full mechanomodule (top) and focused analysis of the N′-AIM–A1–C′-AIM region (bottom), comparing late-stage trajectories [R_5_ (black) vs $R_{5}^{G}$ (gray)]. (g) Intermolecular interaction analysis between AIM and the A1 domain. (h) Steric-clash analysis of A1 shielding by AIM, calculated from interatomic distances <3.0 Å to assess potential GPIbα-binding interference.

The presence of five *N*-linked and nine *O*-linked glycans led to distinct assembly patterns of the VWF mechanomodule (supplementary material Movie S2) and increased the overall hydrodynamic size of VWF [Fig. 2(a)]. To dissect the structural consequences of glycosylation, we performed molecular dynamics trajectory analyses for both glycosylated and non-glycosylated models during the initial (R_1_: 0–100 ns) and final (R_5_: 400–500 ns) stages of simulation (supplementary material Table S3). In the absence of glycans, the mechanomodule showed minimal structural deviation, with an RMSD of 0.26 nm at R_5_ compared to 0.38 nm in the glycosylated model, despite both averaging 0.97 nm during the R_1_ [Fig. 2(b)]. Glycosylation led to a less compact structural organization, with an average radius of gyration (Rg) of 3.75 nm, compared to 3.48 nm in the unglycosylated counterpart [Fig. 2(c)]. This reduced compactness was accompanied by greater solvent exposure, as reflected by a higher solvent-accessible surface area (SASA) of 575.95 vs 523.45 nm^2^ [Fig. 2(d)]. Notably, the number of intramolecular hydrogen bonds (HBs) decreased in the glycosylated form (752 vs 766), indicating weakened interdomain interactions and altered domain packing likely driven by glycan-induced steric hindrance [Fig. 2(e)]. In addition, glycosylation increased the structural flexibility of the mechanomodule, with average Cα atomic fluctuations rising from 0.17 to 0.22 nm [Fig. 2(f)]. Detailed analysis of interdomain (D′D3–A2–A3–A1) and intermodular (N′AIM–A1, C′AIM–A1, and N′AIM–C′AIM) HB and salt bridge (SB) revealed that glycosylation of the VWF mechanomodule attenuates interdomain interactions [supplementary material Fig. S3(A)], while preserving intermodular contacts (AIM–A1) [Fig. 2(g) and supplementary material Fig. S3(B)].

### Electrostatic and steric mechanisms govern autoinhibition by N′AIM and C′AIM

We identified that Glu1260 from the distal N′AIM, along with Glu1264 and Glu1269 from the proximal N′AIM, critically modulate the interaction affinity between N′AIM and the A1 domain. Similarly, Glu1463 of the C′AIM governs its association with A1. Notably, these residues are all negatively charged and form salt bridges with a cluster of positively charged residues on A1, including Arg1274, Arg1306, Arg1308, Arg1312, Arg1315, Arg1334, Arg1336, Arg1341, and Arg1374 [supplementary material Fig. S3(B)]. This charge-based interaction is particularly significant, as GPIbα–A1's physiological binding partner is itself highly negatively charged and engages A1 via these same positively charged regions. These findings suggest that the autoinhibitory mechanisms of both N′AIM and C′AIM are charge dependent, wherein key acidic residues in the AIMs transiently shield the GPIbα-binding site on A1 through electrostatic complementarity, corroborating previous observations.^20^ We observed a correlation between the number of interactions formed between the AIM regions and A1 and their relative autoinhibitory strength. N′AIM, which formed substantially more salt bridges with A1 than C′AIM [supplementary material Fig. S3(B)], demonstrated a greater capacity to sterically occlude the GPIbα-binding site. Remarkably, the presence of eight *O*-linked glycans further amplified the autoinhibitory potential of both N′AIM and C′AIM [Fig. 2(h)], as glycan-induced steric hindrance significantly enhanced their ability to obstruct A1–GPIbα engagement, characterized by the higher number of steric clashes.

**FIG. 3.**
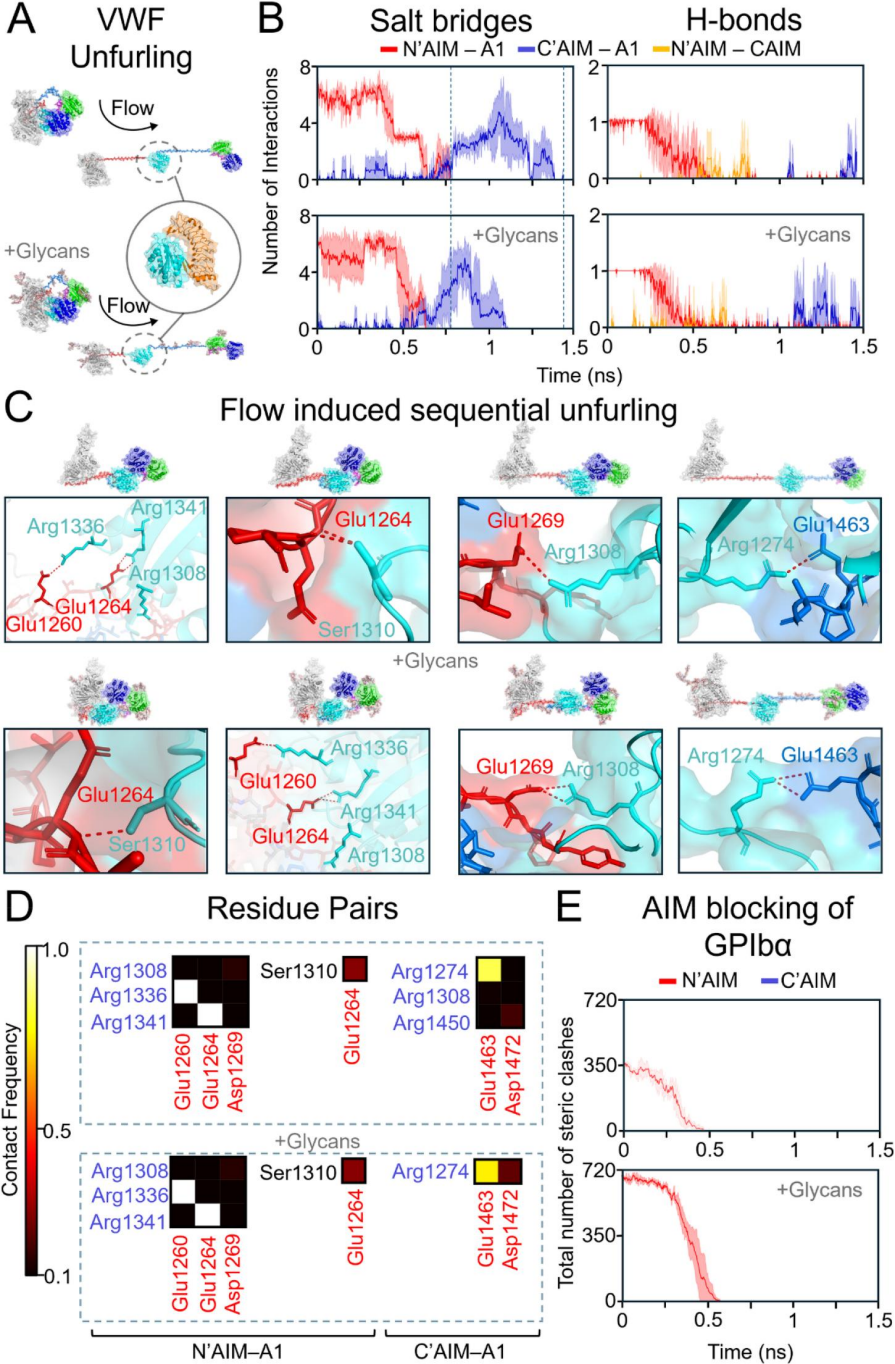
Flow-induced molecular dynamics (flow MD) simulations reveal the unfurling process of the VWF mechanomodule and its regulation of GPIbα accessibility. (a) Force-induced transition of the VWF mechanomodule from a compact “bird's nest” conformation to an extended, uncoiled structure facilitates exposure of the A1 domain for GPIbα binding. (b) Dynamic intermolecular interactions between the A1 domain and the flanking N′AIM and C′AIM regions are illustrated, highlighting their regulatory role during unfolding. Time-resolved interaction profiles are shown as mean ± standard deviation, smoothed using the Savitzky–Golay filter. (c) Trajectory snapshots of key residues involved in modulating N′-AIM and C′-AIM engagement with A1, which together influence the shielding of the GPIbα-binding interface. (d) Contact frequency heatmap showing residue–residue interactions that persist in more than 10% of simulation frames; low-frequency contacts are excluded. Brighter colors indicate higher contact frequencies, while darker regions represent less persistent interactions. (e) Steric-clash analysis of the N′-AIM and C′-AIM regions during unfolding, assessing their capacity to shield the A1 domain from GPIbα through atomic overlap (distance <3.0 Å) smoothed using the Savitzky–Golay filter.

Insights from our flow simulations (supplementary material Movie S3), which recapitulate the flow-induced unfurling of VWF and the uncoiling of N′AIM and C′AIM to expose the A1 domain [Fig. 3(a)], revealed that while *O*-linked glycans enhance steric shielding of A1 from GPIbα, they also modulate the stability of AIM–A1 interactions. Specifically, glycan-induced steric hindrance shortened the lifetimes of both N′AIM–A1 and C′AIM–A1 interactions [Fig. 3(b)], leading to earlier uncoiling events compared to the unglycosylated system [Fig. 3(c)]. Importantly, the key residues mediating these interactions were conserved regardless of glycosylation status [Fig. 3(d)], indicating that the observed differences arise primarily from sterics.^21^ Analysis of steric clashes under flow revealed a progressive reduction in steric obstruction over time, consistent with A1 mechano-presentation [Fig. 3(e)].

**FIG. 4.**
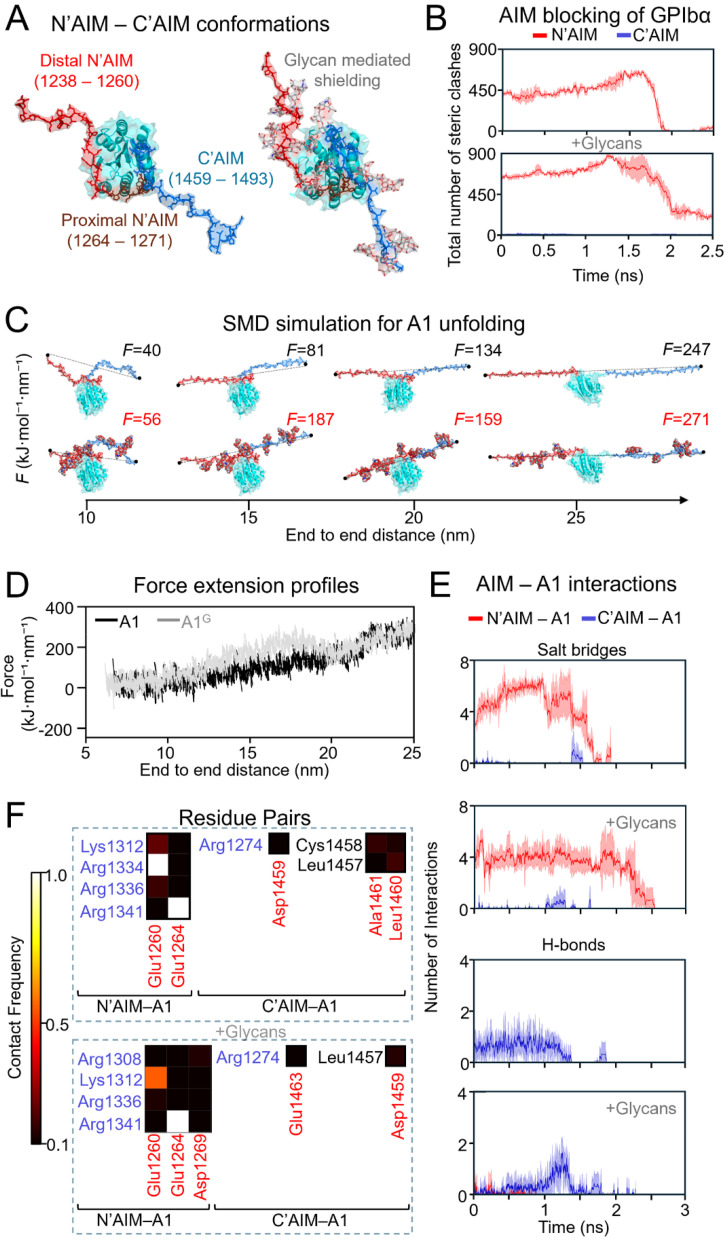
Steered molecular dynamics (steered MD) simulations reveal the force-induced uncoiling of the N′-AIM and C′-AIM regions and their role in A1 shielding under tensile stress. (a) Structural differences in the conformations of N′-AIM and C′-AIM with and without glycans, highlighting the spatial separation between their distal and proximal termini relative to the A1 domain. (b) Steric-clash analysis of the N′-AIM and C′-AIM regions during forced uncoiling, assessing their potential to shield the A1 domain from GPIbα via atomic overlaps (distance <3.0 Å), smoothed using the Savitzky–Golay filter. (c) Representative trajectory snapshots illustrating the force required to uncoil increasing distances between N′-AIM and C′-AIM, in both glycosylated and non-glycosylated contexts. (d) Force–extension profiles showing the mechanical resistance associated with the uncoiling of the AIM regions under steered MD (A1—without glycans; A1^G^—with glycans). (e) Intermolecular interactions between the AIM regions and the A1 domain during force application, revealing transient or persistent binding contacts that may regulate GPIbα accessibility, smoothed using the Savitzky–Golay filter. (f) Contact frequency heatmap showing residue–residue interactions that persist in more than 10% of simulation frames; low-frequency contacts are excluded. Brighter colors indicate higher contact frequencies, while darker colors denote weaker or transient interactions.

To complement our flow MD simulations, which mimic shear forces through solvent-mediated mechanical perturbation, we also conducted steered MD (SMD) simulations (supplementary material Movie S4), applying tensile forces directly through the VWF backbone to assess differences in AIM-mediated shielding of A1. As shown in Fig. 4(a), glycan-induced steric bulk reduced the spatial proximity of both N′AIM and C′AIM to A1, yet these sterics remained effective in obstructing GPIbα access [Fig. 4(b)]. While the steric contribution of C′AIM diminished entirely as unfolding progressed, N′AIM continued to impose steric hindrance, further supporting its dominant role in shielding A1 from GPIbα binding, an observation reported also in previous studies.^15^ Notably, the mean number of steric clashes contributed by C′AIM to GPIbα blocking increased from 0 in the non-glycosylated state to 1.54 with glycans. However, this effect is force-regime dependent: in steered MD, the applied tension pulls C′AIM away from N′AIM, reducing its shielding efficacy. In contrast, flow MD promotes a more distributed uncoiling of N′AIM and C′AIM without backbone-directed tension, allowing C′AIM to maintain closer proximity to A1 for longer durations. This highlights the differential mechanical outcomes imposed by distinct force application paradigms and underscores the context-specific role of AIM in regulating A1 accessibility.

Applying tensile forces to pull the C′AIM away from the N′AIM revealed that a higher force is required in the presence of eight *O*-linked glycans, which likely contribute hydrodynamic drag during extension [Fig. 4(c)]. This results in an increased force threshold needed to achieve equivalent extension distances [Fig. 4(d)]. Despite fewer intermolecular interactions compared to unglycosylated systems [Fig. 4(e)], these contacts persisted for longer durations in glycosylated constructs (supplementary material Fig. S4), with contact frequencies sustained over extended timescales [Fig. 4(e)]. Notably, interactions between N′AIM–A1 and C′AIM–A1 identified in flow simulations were similarly observed in steered MD simulations [Figs. 3(d) and 4(f)], reinforcing the critical role of AIM–A1 affinity, which is predominantly governed by electrostatic forces.

Multiple sequence alignment (MSA) of VWF residues 1238–1493 across *Homo sapiens*, *Mus musculus*, and *Bos taurus* [supplementary material Fig. S5(A)] revealed that the proximal N′AIM is significantly more conserved than the distal N′AIM, highlighting its evolutionary and physiological importance. Notably, the distal N′AIM showed no sequence consensus in *B. taurus*, further emphasizing its species-specific variability. Among all identified glycosylation sites, only the *O*-linked glycans at Ser1263 and Thr1487 were not conserved. Integrating these sequence insights with our flow and steered MD simulations, we observed that proximal N′AIM acts as the final barrier, maintaining steric hindrance within 3.0 Å of GPIbα. Furthermore, the glycan at Ser1263 emerged as the last remaining glycan obstructing A1 from GPIbα engagement, reinforcing its functional importance in autoinhibition [supplementary material Fig. S5(B)].

### Mechanical unmasking of GPIbα-binding sites by the VWF D′D3 region

**FIG. 5.**
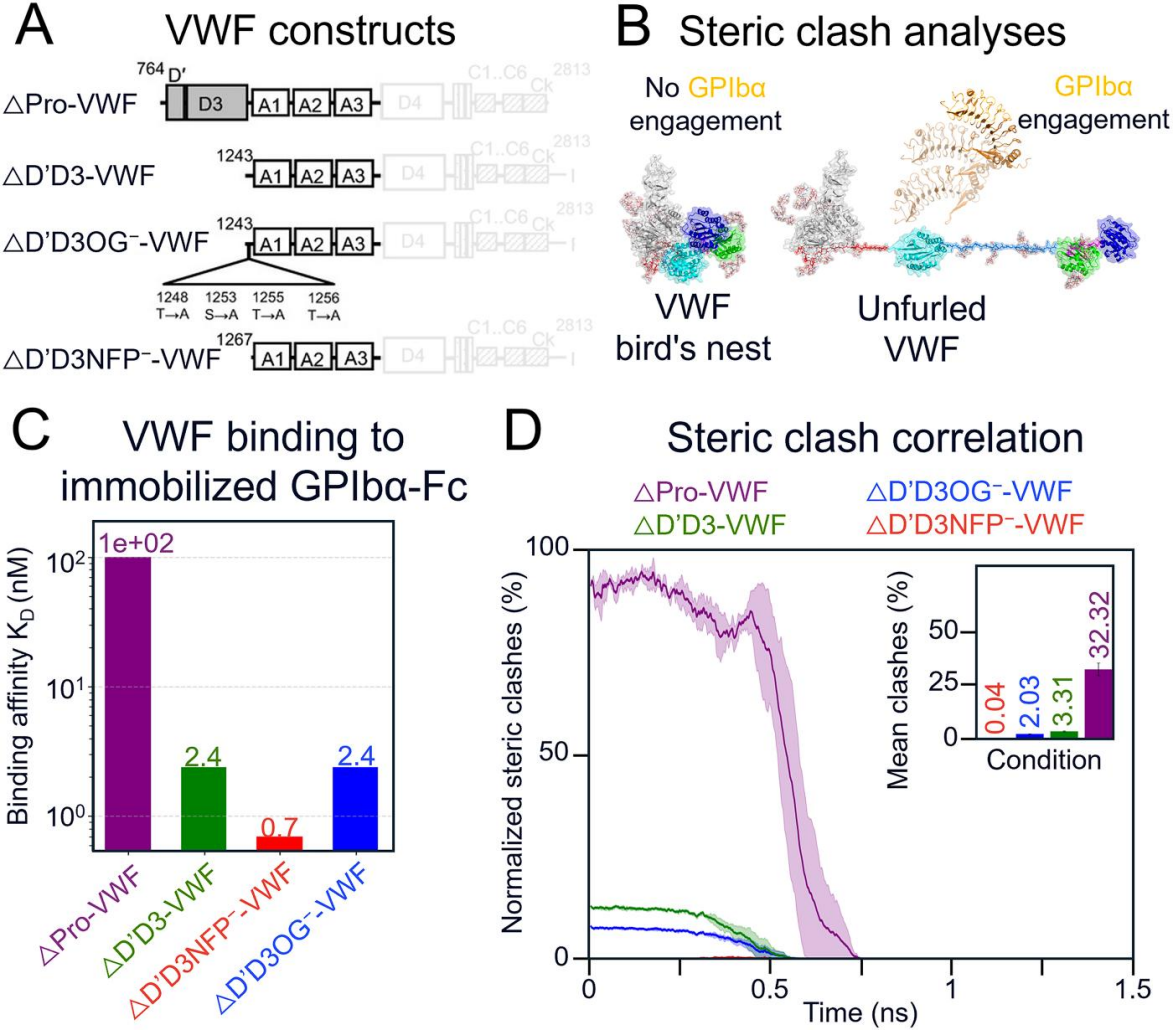
Kinetic correlation landscape of VWF–GPIbα affinity. (a) VWF constructs examined in Madabhushi *et al.*^22^ (b) Schematic of the steric-clash analysis used to evaluate GPIbα binding to VWF during force-induced unfolding. (c) Experimentally measured binding affinities reported by Madabhushi *et al.*^22^ (d) Normalized steric clash between GPIbα and distinct VWF regions during unfolding, smoothed using a Savitzky–Golay filter. The inset shows the mean steric-clash values.

To further corroborate the unfolding kinetics of VWF observed in our simulations, we leveraged previously published kinetic data from Madabhushi *et al.*^22^ examining multiple VWF constructs, including ΔPro-VWF, ΔD′D3-VWF, ΔD′D3NFP^−^-VWF, and ΔD′D3OG^−^-VWF [Fig. 5(a)]. Although these studies employed dimeric VWF constructs, we established correspondence between experiment and simulation by comparing the reported dissociation constants (K_d_) with steric-clash and accessibility analyses of GPIbα binding during force-induced unfolding in our flow-based molecular dynamics simulations [Fig. 5(b)]. Madabhushi *et al.* demonstrated that deletion of the D′D3 domain together with the N-terminal flanking peptide up to residue 1267 resulted in the highest binding affinity for GPIbα^22^ [Fig. 5(c)], highlighting the critical shielding role of these regions. Consistent with these observations, our simulations reproduced the same affinity trend, revealing increased GPIbα accessibility upon force-mediated unmasking of these domains [Fig. 5(d)]. Collectively, our results demonstrate that flow-induced simulations can identify mechano-presented epitopes and delineate mechanically protected vs exposed regions of VWF, providing a mechanistic framework to guide the rational design of next-generation mechanomedicine.

## DISCUSSION

VWF undergoes dramatic conformational transformations, transitioning from a compact “bird's nest” architecture to an elongated, uncoiled state in response to shear flow.^5^ Although neural network-based models have revolutionized the prediction of static protein structures, they remain inherently limited in capturing the dynamic flexibility and structural heterogeneity that define mechanosensitive proteins such as VWF.^23^

To transcend these limitations, molecular dynamics (MD) simulations have become indispensable, offering atomistic resolution into von Willebrand factor (VWF) behavior under tensile and flow conditions.^24–28^ To contextualize our simulation parameters, we mapped the applied forces to physiological shear stress, as detailed in Sec. SI of the supplementary material. Arterial shear stress typically ranges from 10 to 70 dyn/cm^2^, but can exceed 400 dyn/cm^2^ in stenotic or injured arterioles. Experimental studies have shown that VWF undergoes significant shear-dependent extension at values up to 1280 dyn/cm^2^.^29^ Using the Newtonian fluid relation $\tau =\mu \dot{\gamma }$,^30^ where μ is the dynamic viscosity of water at 310 K (0.693 mPa s), we estimated the corresponding shear rates as ∼1.01 × 10^4^ s^−1^ for arterial baseline and ∼1.85 × 10^5^ s^−1^ for pathological peak conditions. In our flow MD setup, the nominal shear rate is defined by the velocity gradient $\dot{\gamma }=v/L$, where v is the pulling velocity and L is the characteristic solvent box length (150 nm). The selected pull rate of 0.01 nm ps^−1^ corresponds to an accelerated regime approximately 360 times higher than the estimated pathological shear rate. While these effective flow rates exceed physiological conditions, a common requirement in atomistic MD to access rare events within accessible timescales. The flow MD setup is calibrated to preserve structural integrity and correctly rank the relative kinetic stability of VWF domains. These simulations provide mechanistic insight into VWF mechano-modulation, revealing how mechanical forces regulate conformational transitions and ultimately influence biological function.^23^

We selected to simulate the D′D3–A3 mechanomodule due to prior use in kinetic studies^31^ where spatial segregation of A1 domains was critical. Our choice for restraining the Cα atoms of the D′D3 domain during the flow was attributed to previous studies as mentioned in Sec. SII of the supplementary material. Importantly, this construct retains the *O*-linked glycans and structural motifs proposed to regulate the accessibility and functional presentation of the A1 domain.^31^ Our free, flow-based, and steered MD simulations not only substantiated previous experimental findings but also revealed previously unrecognized mechanisms of VWF mechanomodulation, including dynamic interactions between the N′AIM and C′AIM regions and the A1 domain.

It is well established that glycans enhance the shielding of the A1 domain from GPIbα. Our steric-clash analyses not only recapitulated this phenomenon but further demonstrated that the N′AIM region alone is sufficient to inhibit A1–GPIbα binding [Figs. 2(h), 3(e), and 4(b), and supplementary material Table S4], corroborating previous studies.^14,15^ Furthermore, we demonstrate that the interactions between N′AIM and the A1 domain are greater than those of C′AIM–A1 [Figs. 2(g), 3(d), and 4(f), and supplementary material Tables S5 and S6], corroborating earlier findings.^4^ This enhanced affinity is primarily driven by electrostatic interactions, including the formation of salt bridges, which contribute to the superior autoinhibitory capacity of N′AIM. Our steered MD simulations also corroborate previous findings that glycosylated forms of VWF exhibit greater mechanical stability and require higher forces to induce unfolding.^32^

Interestingly, our simulations did not capture direct interactions between the distal regions of N′AIM and C′AIM, as previously proposed.^13^ This discrepancy likely stems from a key difference in simulation setup: we modeled A1 in the context of its flanking domains, D′D3 and A2, which impose spatial constraints that may hinder distal N′AIM–C′AIM interactions. In contrast, prior studies that simulated isolated N′AIM–A1–C′AIM constructs without neighboring domains have reported contacts between the distal ends of these autoinhibitory modules.^33^ These observations suggest that the conformational pose and interaction landscape of N′AIM and C′AIM are strongly influenced by their structural context and that domain architecture plays a critical role in regulating A1 autoinhibition.^34^

Our simulations, complemented by MSA, reveal key regions within the N′AIM and critical glycans that act as the final steric barriers to GPIbα engagement during VWF extension. Prior studies have suggested that interactions between the proximal N′AIM and the A1 domain are essential for maintaining A1 in an autoinhibited conformation; disruption of these interactions is thought to activate A1, priming it for GPIbα binding.^13^ Our simulations identify specific residues, most notably Glu1260 within the proximal N′AIM, Glu1264 within the distal N′AIM, and Glu1463 from the C′AIM, that regulate AIM–A1 interactions [Figs. 3(d) and 4(f), and supplementary material Fig. S3(B)]. To ensure these interactions were independent of the starting orientation and flow direction, we performed ensemble simulations across multiple orientations, as described in Sec. SIII of the supplementary material. MSA further revealed that the *O*-linked glycosylation site at Ser1263 is not conserved across species. In contrast, the proximal region of N′AIM, but not the distal segment, exhibited relatively high sequence conservation, suggesting a potential role of these regions in serving as the final shield of A1 from premature GPIbα engagement (supplementary material Fig. S5).

These findings underscore the utility of our analyses in elucidating VWF dynamics under both static conditions and flow-induced activation. Our approach provides atomic-level resolution to identify epitopes that are shielded in the quiescent state and selectively exposed under force. This paradigm is critical for rational drug design, enabling the prioritization of mechano-presented epitopes over those accessible in static structures, thereby facilitating efficient preclinical assessment prior to microfluidic experimentation and downstream validation.

## METHODS

### *In silico* modeling of the VWF mechanomodule

To model the VWF mechanomodule in a physiologically relevant state and interrogate mechano-presentation, particularly the autoinhibitory roles of the N′AIM and C′AIM of A1, we constructed an integrative structural model spanning residues 764–1873 (D′D3–A3 domains). Initial structural predictions were generated using AlphaFold 3,^10^ yielding a model with moderate interdomain confidence (predicted TM-score ≈0.482). To improve structural accuracy, we developed a hybrid modeling pipeline combining deep-learning-based predictions with high-resolution crystallographic templates. Linker regions absent in crystal structures [Fig. 1(c)] were reconstructed using ColabFold (CF), an accelerated AlphaFold 2 implementation with MMseqs2^35,36^ for multiple sequence alignment. Experimentally resolved structures for D′D3 (PDB ID: 6N29), A1 (PDB ID: 1SQ0), A2 (PDB ID: 3GXB), and A3 (PDB ID: 4DMU)^37–40^ were aligned to the CF scaffold. CF-predicted domains were replaced with their crystal counterparts to maintain empirical accuracy [Fig. 1(d)], as AlphaFold predictions, although reliable, cannot supersede experimentally validated coordinates.^41^ The hybrid CF + SM model was assembled using PyMOL^42^ and SWISS-MODEL (SM)^43^ using the user template option, followed by energy minimization in Swiss-PdbViewer^44^ to remove steric clashes and optimize stereochemistry. Model validation against AlphaFold 3 and experimental templates was performed using TM-score, RMSD, and MolProbity^45^ metrics. VWF contains five *N*-linked and nine *O*-linked glycosylation sites [Fig. 1(e), left]. Structural models of glycans, primarily disialylated core-1 *O*-glycans (∼70%) and monosialylated bi-antennary *N*-glycans (∼80%)^46^ [Fig. 1(e), right], were generated using the Glycan Reader & Modeler in CHARMM-GUI^47^ and incorporated into the final atomistic model.

### Molecular dynamics simulation of VWF mechano-presentation: From compact to extended states

#### Equilibrium (free) MD simulations—The “bird's nest” conformation

All molecular dynamics (MD) simulations were performed using GROMACS 2021.2^48^ with the CHARMM27 force field and TIP3P water model. All systems were neutralized by adding counter Na^+^ and Cl^−^ ions using the genion tool. Systems were energy-minimized with the steepest descent algorithm and equilibrated in the NVT and NPT ensembles (100 ps each) at 300 K, using a 2 fs time step and the leap-frog integrator. Production simulations of 100 ns employed particle-mesh Ewald (PME) for long-range electrostatics and optimized nonbonded cutoffs following Lemkul.^49^ Simulations with diverging backbone RMSD profiles were iteratively refined by restarting from the final frame until convergence was achieved. The same procedure was applied to glycosylated models.

#### Flow MD simulations—Solvent-driven unfolding of the mechanomodule

Flow was applied by pulling a 5-nm slab of water oxygen atoms along the Z axis using a harmonic spring potential (1000 kJ mol^−1^ nm^−2^) at a constant velocity of ∼0.01 nm ps^−1^. This setup, commonly referred to as Constant Velocity Steered Molecular Dynamics (cv-SMD), allows the water slab to be “pulled” through the periodic box, creating a steady-state flow where the applied force dynamically responds to the system's internal resistance (Herrera-Rodríguez *et al.*,^24^ Zenodo DOI: 10.5281/zenodo.2538871). The applied force exceeds physiological shear rate values, but such accelerated conditions are standard in molecular dynamics to observe unfolding events within accessible simulation timescales. This setup enabled controlled, stepwise uncoiling of VWF and characterization of its mechano-presentation under flow (supplementary material Movie S1). Simulations were conducted in triplicate for the mechanomodule both with and without glycans to investigate unfolding patterns, the influence of interdomain interactions, and the role of AIM in exposing A1. To isolate solvent-induced effects, the Cα atoms of the D′D3 domain were restrained with a harmonic potential (1000 kJ mol^−1^ nm^−2^) in all simulations, preventing translational drift.

#### Steered MD simulations—Glycan-mediated mechanical resilience

Steered MD (SMD) simulations were performed to probe glycan-mediated stabilization of A1, following established protocols.^28^ Each system was embedded in a 10 × 200 × 10 nm box with CHARMM36 protein/carbohydrate parameters and TIP3P water using CHARMM-GUI.^47^ A harmonic spring (1000 kJ mol^−1^ nm^−2^) was applied between the Cα atoms of Asn1493 and Gln1238, pulling along the Y axis at 0.01 nm ps^−1^ to mimic atomic force microscopy or optical tweezer setups.^50^ Force–extension profiles were recorded, and rupture forces were defined as the peak force prior to detachment. Triplicate simulations were averaged to quantify glycan effects.

### Data analysis and visualization

Structural and dynamical analyses were conducted using GROMACS tools: gmx rms (RMSD) and gmx rmsf (root mean square fluctuation), gmx gyrate (radius of gyration), gmx sasa (solvent accessibility), and gmx hbond (hydrogen bonding).^51,52^ Trajectories were sorted using MDTraj^53^ and visualized in PyMOL, VMD, and QtGrace.^42,54,55^ The structural quality of the AlphaFold 3 model, the ColabFold–SWISS-MODEL hybrid (CF + SM), and the 500-ns simulation-refined CF + SM structure was assessed using MolProbity, providing comprehensive all-atom validation through integrated metrics including clashscore, rotamer outliers, and Ramachandran statistics.^56^

Intermolecular interactions and steric clashes were analyzed using MDAnalysis v 2.0.^57^ Hydrogen bond (HB) and salt bridge (SB) interactions were evaluated from the MD trajectories. For H-bonds, donor atoms were defined as nitrogen or oxygen atoms capable of forming hydrogen bonds, specifically N, NE, ND1, NE2, NH1, NH2, OG, OG1, and OH; acceptor atoms were defined as O, OD1, OD2, OE1, OE2, OG, OG1, and OH. An HB was considered present when the donor–acceptor distance was ≤3.5 Å and the angle between donor and acceptor atoms (approximating the donor–hydrogen–acceptor angle) was ≥150°, following established geometric guidelines. SB was defined as interactions between oppositely charged side chains, specifically between the positively charged nitrogen atoms of Lys (NZ) and Arg (NE, NH1, and NH2) and the negatively charged oxygen atoms of Asp (OD1 and OD2) and Glu (OE1 and OE2). An SB was considered present when the distance between any such atom pair was ≤3.5 Å. Analyses were performed over every frame of the simulation trajectory, and interaction frequencies were recorded for quantitative comparison. Intermolecular interactions were analyzed by combining data from three independent replicate simulations. Each replicate's trajectory frames were reindexed and concatenated to form a continuous dataset, allowing comprehensive assessment across all simulations. Contact frequencies were calculated as the fraction of frames in which each residue pair was observed to form an SB or HB across the concatenated dataset. To focus on persistent interactions, only contacts present in at least 10% of the combined frames were retained for further analysis and visualization as a heatmap.

MD trajectories were analyzed against a static reference structure containing the GPIbα domain (PDB ID: 1SQ0) to quantify potential steric clashes. The static A1 domain from the reference structure was aligned to each frame of the trajectory using the heavy atoms of residues 1290–1430 as the alignment region. Following alignment, interatomic distances were computed between heavy atoms of the static GPIbα domain (residues 1–265) and dynamic regions of interest in the trajectory: NAIM (residues 1238–1271) and CAIM (residues 1459–1493), including any *N*- or *O*-linked glycans within 5 Å of each region adapted from previous studies.^15^ Steric clashes were defined as any heavy atom pairs within 3.0 Å between the static reference and the dynamic trajectory. Clash counts were computed on a per-frame basis to enable quantitative assessment of glycosylation-dependent interference with GPIbα binding. Multiple sequence alignments (MSAs) of the A1 domain from *H. sapiens (NCBI Reference: AAB34053.1)*, *M. musculus (NCBI Reference: ABC86574.1)*, and *B. taurus (NCBI Reference:* NP_*001192237.1)* were performed, and residue conservation was analyzed using Jalview.^58^

### Limitations

Although our simulations provide a novel approach to understanding VWF unfolding kinetics, with corroboration from prior experimental studies, they are inherently limited by the scope of existing experimental data. Future studies examining the effects of VWF glycosylation and its differential interactions with therapeutic agents will be essential to validate and complement our in *silico* observations with experimental benchmarks.

## SUPPLEMENTARY MATERIAL

See the supplementary material for Figs. S1–S9, Tables S1–S7, Movies S1–S7, and Secs. SI–SIII. Section SI describes the flow simulation parameters. Section SII details the protein restraints applied in the flow simulations. Section SIII presents the multi-orientation flow simulation analyses.

## Data Availability

The data that support the findings of this study are openly available in Zenodo at https://doi.org/10.5281/zenodo.19656129, Ref. 59 and GitHub at https://github.com/NaveenEugeneLouis/Flow-molecular-dynamics-simulations-of-von-Willebrand-factor-.git, Ref. 60.
